# Exploring Aspirin’s Potential in Cancer Prevention: A Comprehensive Review of the Current Evidence

**DOI:** 10.7759/cureus.70005

**Published:** 2024-09-23

**Authors:** Carla Isabella Miret Durazo, Stephin Zachariah Saji, Akash Rawat, Ada L Motiño Villanueva, Amit Bhandari, Tutut Nurjanah, Niharika Ryali, Ismael Germán Zepeda Martínez, Josue A Cruz Santiago

**Affiliations:** 1 General Practice, Centro de Estudios Universitarios Xochicalco, Escuela de Medicina Tijuana, Tijuana, MEX; 2 General Medicine, Our Lady of Fatima University, Valenzuela, PHL; 3 Department of General Medicine, Himalayan Institute of Medical Sciences, Swami Rama Himalayan University, Dehradun, IND; 4 Department of General Medicine, Universidad Católica de Honduras, Tegucigalpa, HND; 5 Internal Medicine, American University of the Caribbean School of Medicine, Cupecoy, SXM; 6 Department of General Medicine, Universitas Yarsi, Jakarta, IDN; 7 Department of General Medicine, Gandhi Medical College, Hyderabad, IND; 8 Faculty of Medicine, Universidad Nacional Autónoma de México, Ciudad de México, MEX; 9 Department of General Medicine, Universidad Autónoma de Guadalajara, Guadalajara, MEX

**Keywords:** adverse effects, aspirin, breast cancer, cancer prevention, colon cancer, prostate cancer

## Abstract

Aspirin, traditionally recognized for its analgesic, anti-inflammatory, antipyretic, and antiplatelet effects, has recently attracted attention for its potential role in cancer prevention. Initially studied for cardiovascular disease prevention, emerging evidence suggests that aspirin may reduce the risk of certain cancers, particularly colorectal cancer (CRC). This narrative review integrates findings from early studies, animal models, epidemiological data, and clinical trials to evaluate aspirin's efficacy as a chemopreventive agent. Aspirin’s anticancer effects are primarily attributed to its cyclooxygenase (COX) enzyme inhibition, which decreases prostaglandin E2 (PGE2) levels and disrupts cancer-related signaling pathways. While epidemiological studies support an association between aspirin use and reduced cancer incidence and mortality, especially for CRC and potentially for breast (BC) and prostate cancers (PCa), the risk of adverse effects, such as gastrointestinal (GI) and intracranial bleeding, complicates its use and warrants careful consideration. The decision to use aspirin for cancer prevention should be individualized, balancing its therapeutic benefits against potential adverse effects. It also underscores the necessity for further research to refine dosage guidelines, assess long-term impacts, and explore additional biomarkers to guide personalized cancer prevention strategies.

## Introduction and background

Aspirin is one of the oldest medications and is still widely used today. The modern history of aspirin's precursors, known as salicylates, began in 1763 with Reverend Stone, who first described their antipyretic effects. This development continued into the 19th century with these compounds' extraction and chemical synthesis, culminating in Bayer (Bayer AG, Leverkusen, North Rhine-Westphalia, Germany) chemist Felix Hoffmann's synthesis of aspirin in 1897 [1]. Aspirin is a versatile medication commonly used as an analgesic, anti-inflammatory, antipyretic, and antiplatelet agent [2,3]. Over the years, aspirin has played a significant role in preventing cardiovascular diseases [4]. Recently, scientific interest has shifted toward understanding its potential role in cancer prevention. It is thought that aspirin might interfere with cancer pathways, including proliferation, metastasis, thrombosis, and DNA repair [5]. 

In 2022, there were an estimated 20 million new cancer cases and 9.7 million cancer-related deaths. The estimated number of individuals alive five years after a cancer diagnosis was 53.5 million. Approximately one in five people will develop cancer in their lifetime, with about one in nine men and one in 12 women dying from the disease [6]. Despite extensive research over many years, the precise mechanisms by which aspirin may contribute to cancer prevention remain unclear, and the findings continue to be a subject of ongoing debate. Some studies claim that long-term aspirin use in individuals without a history of cancer is not associated with a significant reduction in total cancer incidence, cancer mortality, or overall mortality [7], or it could even cause an adverse effect [8]. Conversely, studies with promising methodological approaches, such as meta-analyses, suggest that aspirin use is associated with a reduced risk of several types of cancer [9-12]. Given the extensive research on aspirin and its broad application in clinical practice due to its therapeutic properties, this narrative review aims to explore and synthesize the current evidence on aspirin's role in cancer prevention.

## Review

Early evidence of aspirin’s anticancer properties

The investigation into aspirin's potential anticancer properties has become a significant area of study due to its widespread use and the intriguing findings that have emerged over the years. A case-control study explored the association between colorectal cancer (CRC) risk and various chronic diseases, surgeries, and medications, analyzing 715 patients with CRC and 727 controls [13]. This study found a statistically significant reduction in cancer incidence among patients who consumed aspirin and aspirin-containing medications, a consistent trend for both men and women.

Early research supports the hypothesis that regular aspirin use may reduce the risk of fatal colon cancer [14]. However, whether this is due to a direct effect of aspirin, possibly mediated by the inhibition of prostaglandin synthesis, or other factors indirectly associated with aspirin use remains unclear. Building on these initial observations, subsequent research has reinforced that aspirin could be crucial in cancer prevention. The initial hypothesis that platelet function influences cancer progression led to investigations into aspirin's effects on metastatic processes [15].

Aspirin’s mechanism of action in cancer

Aspirin exerts its anticancer effects primarily through the inhibition of cyclooxygenase enzymes (COX-1 and COX-2), which are crucial for converting arachidonic acid into prostaglandins (PGs) like prostaglandin E2 (PGE2). By irreversibly inhibiting COX-2, aspirin reduces PGE2 levels, thereby decreasing the activation of cancer-related signaling pathways such as PI3K/AKT (phosphatidylinositol 3-kinase/protein kinase B) and ERK and promoting apoptosis in cancer cells ​[16,17]. Additionally, COX-2 is often overexpressed in various cancers, enhancing cancer cell invasiveness and angiogenesis [18]. This overexpression has been linked to the progression of several cancers, including breast cancer (BC), prostate cancer (PCa), and esophageal carcinoma.

The inhibition of COX-2 by aspirin also impacts other molecular pathways. For example, oncogenic RAS gene expression in epithelial cells can induce COX-2, further activating pathways like MAPK, which leads to the phosphorylation of transcription factors such as AP-1 and NF-κB ​[19]. These transcription factors enhance COX-2 expression, supporting cancer cell proliferation and inflammation, further exacerbating tumor progression. In PCa, protein kinase C (PKC) overexpression similarly activates COX-2 and its associated transcription factors, reinforcing the inflammatory environment conducive to cancer development ​[20,21].

Beyond COX inhibition, aspirin reduces the production of inflammatory cytokines, chemokines, and growth factors that promote cell proliferation and inhibit apoptosis. Chronic inflammation, which can cause DNA damage through reactive oxygen species (ROS), contributes to carcinogenesis ​[22]. By mitigating these inflammatory processes, aspirin also decreases cell proliferation, angiogenesis, and metastasis. Moreover, aspirin inhibits thromboxane A2 (TXA2) and PGE2, counteracting platelet activation, which plays a role in tumor cell survival, dissemination, and immune evasion. This inhibition reduces the recruitment of platelet-derived growth factors (PDGF) and interleukin 1β, potentially slowing tumor growth and progression [23].

Recent evidence also suggests that aspirin’s anticancer effects extend beyond COX pathways. For instance, aspirin inhibits IκB kinase (IKK), preventing NF-κB activation and reducing cell survival signals [18]. It also activates AMPK, indirectly inhibiting mTOR signaling, critical for cell growth and proliferation, thus inducing apoptosis [24,25]. Additionally, aspirin modulates Wnt signaling, which influences tumorigenesis along with COX-2/PGE2 pathways ​[26].

Furthermore, aspirin helps reduce cancer metastasis by inhibiting platelet-tumor cell interactions, enhancing immune surveillance, and suppressing inflammatory and COX-2 pathways. This multifaceted mechanism of action positions aspirin as a promising adjunct in cancer therapy, particularly in preventing metastasis ​[23]. For example, studies have shown that reducing the number of host platelets before tumor inoculation in mice decreased the number of metastases, suggesting that aspirin's effects on platelet function may be critical in limiting cancer spread [15].

Animal and cellular studies

Transgenic mouse studies have demonstrated aspirin’s efficacy in combating various cancers, showing reduced tumor growth following treatment [27]. Evidence indicates that daily low-dose aspirin is beneficial in reducing inflammation in mice with neuroblastoma. In one study, tyrosine-hydroxylase-MYCN (TH-MYCN) homozygous mice were monitored for tumor-induced inflammation and growth in vivo. Administration of 10 mg of aspirin significantly decreased tumor cells in the innate immune system and reduced inflammatory mediators such as TXA2 and PGD2, ultimately lowering the overall tumor burden [27]. Similarly, another study examined low-dose aspirin’s effects on colon tumor development in mice. Researchers found that a dose of 25 mg/kg/day, roughly equivalent to 75-100 mg in humans, could inhibit TXA2 formation in plasma and serum, thereby reducing cancer-associated inflammation [28]. By the end of the study, aspirin treatment had reduced the total number of tumors [28]. These findings highlight the benefits of low-dose aspirin in mitigating tumor-induced inflammation and spread.

Research has also revealed that COX-independent mechanisms contribute to the beneficial impact of non-steroidal anti-inflammatory drugs (NSAIDs) on tumorigenesis. Notably, a study found that the potency of NSAIDs in inhibiting COX-1 and COX-2 does not always correlate with their effectiveness in inhibiting tumor growth [29]. The dose required to restrict tumor growth is often much higher than that needed to inhibit COX, emphasizing the importance of non-COX mechanisms in stunting tumor growth [29].

An interesting mechanism that doesn’t involve COX is related to NSAID metabolites like sulindac sulfone. While it doesn’t inhibit COX, it effectively slows tumor growth. When converted to its active form, sulindac sulfide, it further prevents tumor development and causes cancer cell death in different organs [29]. This indicates that aspirin might reduce tumor size through ways other than COX inhibition. While mouse models provide valuable insights into drug effects and mechanisms, their extrapolation to human outcomes is not without limitations. One significant constraint is the difference in metabolic and physiological responses between mice and humans. For instance, while aspirin may exhibit similar anti-inflammatory effects in both species, the specific biochemical pathways involved can vary [24].

Overview of epidemiological studies

Recent epidemiological studies extensively explore the association between regular aspirin use and cancer prevention [30]. These studies evaluate aspirin’s long-term effects as a chemopreventive agent in populations with varying cancer risks. They typically involve large cohort studies, case-control studies, and population-based registries, focusing on cancers such as CRC, BC, PCa, esophageal cancer, pancreatic cancer, hepatocellular carcinoma (HCC), and cholangiocarcinoma. The diversity in studied populations, across age, gender, ethnicity, and health conditions, allows for a nuanced understanding of aspirin's effects. However, separating the influence of confounding variables, such as diet, lifestyle, and genetic predispositions, remains challenging.

Evidence shows that aspirin effectively lowers the risk of CRC, with results comparable to established screening methods like fecal occult blood testing (FOBT) and flexible sigmoidoscopy [31]. Aspirin is particularly beneficial for reducing cancer risk in the proximal colon, whereas flexible sigmoidoscopy is more effective for the distal colon [31]. This difference arises because aspirin’s anti-inflammatory effects target cancer mechanisms prevalent in the upper colon. Meta-analyses of both cohort studies and randomized controlled trials reveal that aspirin use significantly lowers CRC risk [32]. Risk reduction is substantial for those using aspirin long-term, but benefits decline after discontinuation. Evidence suggests that patients who recently stopped taking aspirin experience a more marked risk reduction compared to those who discontinued it some time ago [33]. Post-diagnostic aspirin use has significantly reduced CRC mortality, while pre-diagnostic use has had less impact on mortality reduction [34]. Additionally, double-blind, randomized clinical trials have shown that daily aspirin added to therapy significantly reduced the incidence of CRC in high-risk individuals with Lynch syndrome over 10 years, with 20 years of follow-up data reinforcing its long-term benefit in cancer prevention for this population [35].

Aspirin’s potential benefits extend to other cancers, including esophageal, BC, and PCa. For BC, regular aspirin use may lower the risk of developing the disease. Both animal and observational studies suggest that aspirin may contribute to reduced incidence, increased survival rates, and slower tumor progression. A 2016 meta-analysis found that women who regularly took aspirin had a lower incidence of BC compared to non-users [36]. This effect relates to aspirin’s impact on estrogen metabolism, influencing hormonal pathways involved in BC development and potentially decreasing risk by altering estrogen levels and activity [32,37,38]. In terms of esophageal cancer, aspirin shows promise in reducing the risk of both esophageal cancer and Barrett’s esophagus. However, it has not been shown to improve survival in existing cases, possibly due to limited power and a lack of post-diagnosis analysis [39-41]. For pancreatic cancer, aspirin for at least five years significantly reduces the risk of death, though this protective effect becomes apparent only after a five-year lag period [39]. Research indicates that aspirin lowers the risk of developing PCa and may reduce disease progression and recurrence. A meta-analysis has also revealed that regular aspirin users have a reduced risk of PCa and less severe forms of the disease [42]. These findings are significant for patients already diagnosed with PCa, as aspirin may aid in managing long-term disease progression.

HCC, a major type of liver cancer, is potentially impacted by aspirin due to its inhibition of COX-1 and COX-2. This inhibition may help reduce chronic inflammation and cellular damage associated with HCC development. While several studies suggest a protective effect of aspirin against HCC, with varying degrees of risk reduction, the exact relationship remains complex. It is influenced by dosage, duration of use, and underlying liver conditions [43-45]. Similarly, primary sclerosing cholangitis (PSC) is associated with an increased risk of developing cholangiocarcinoma, a challenging complication that complicates patient management [46]. To address this risk, ongoing clinical trials, such as the Asp-PSC trial, are investigating the impact of aspirin on PSC outcomes [47]. The Asp-PSC trial aims to assess whether aspirin can effectively reduce the incidence of cholangiocarcinoma and improve overall outcomes in PSC patients. This study seeks to leverage aspirin's anti-inflammatory properties to alter the disease trajectory and enhance patient survival. Detailed characteristics and findings of the most recent and larger studies are summarized in Table 1 [32,34,38-40,42,48,49], providing a clear snapshot of aspirin’s efficacy across different cancer types and study designs.

**Table 1:** Summary of major epidemiological studies on aspirin and cancer prevention BC: Breast cancer; CRC: Colorectal cancer; HCC: Hepatocellular carcinoma; HR: Hazard ratio; PCa: Prostate cancer; PIK3CA: Phosphatidylinositol-4,5-bisphosphate 3-kinase catalytic subunit alpha; PTGS2: Prostaglandin-endoperoxide synthase 2; RCT: Randomized controlled trials; RR: Relative risk

Author	Year	Study type	Cancer type	Sample size	Aspirin use duration	Cancer risk	Mortality	Key points
Wang et al. [32]	2021	Meta-analysis	Colorectal cancer	2,037,666	Five years	RR 0.85 (95% CI: 0.78-0.92)	RR 0.63 (95% CI: 0.49-0.80)	Aspirin use has been associated with a reduced risk of developing cancer risk.
Madge et al. [34]	2022	Meta-analysis	Colorectal cancer	237,245	Eight years	NA	HR 0.74 (95% CI: 0.62–0.89)	Aspirin may improve survival in CRC patients, especially those with PIK3CA mutations or high PTGS2 expression.
Wang et al. [32]	2021	Meta-analysis	Breast cancer	2,037,666	Five years	RR 1.03 (95% CI: 0.98-1.09)	RR 0.81 (95% CI: 0.65-1.00)	Post-diagnostic aspirin use was identified to be significantly associated with a reduced risk of BC mortality.
Liu et al. [38]	2021	Meta-analysis	Breast cancer	142,644	NA	NA	HR 0.69 (95% CI: 0.61–0.76)	Aspirin use may improve all-cause mortality, specific mortality, and risk of recurrence/metastasis in patients with BC.
Ma et al. [48]	2023	Meta-analysis	Prostate cancer	107,034,535	Five years	RR 0.96 (95% CI: 0.95–0.98)	RR 0.88, (95% CI: 0.82–0.95)	There is an independent correlation between the use of aspirin and reductions in both the incidence and mortality rates of PCa. The impact of aspirin on PCa occurrence was found to be dependent on both dosage and duration.
Huang et al. [42]	2014	Meta-analysis	Prostate cancer	54,929	Four years or more	RR 0.86 (95% CI: 0.81–0.92)	NA	Findings suggest that regular use, especially long-time regular aspirin use, may reduce the risk of overall and advanced PCa.
Bosetti et al. [40]	2020	Meta-analysis	Esophageal cancer	6211	Eight years	RR 0.67 (95% CI: 0.57-0.79)	NA	Aspirin use is associated with a reduced risk of common cancers, based on evidence from cohort studies and RCTs.
Spence et al. [39]	2018	Cohort	Esophageal cancer	4654	Five years	NA	HR 1.03 (95% CI, 0.85–1.2)	Low-dose aspirin use does not improve survival in patients with esophageal or gastric cancer based on two independent population-based cohorts.
Bosetti et al. [40]	2020	Meta-analysis	Pancreatic cancer	12,193	Eight years	RR 0.78 (95% CI: 0.68-0.89)	NA	Findings suggest that there is a reduced risk of pancreatic cancer risk.
Simon et al. [49]	2020	Meta-analysis	Hepatocellular carcinoma	50,275	10 years	HR 0.67 (95% CI: 0.61-0.73)	HR 0.70 (95% CI: 0.63-0.75)	The use of low-dose aspirin was associated with a significantly lower risk of HCC and lower liver-related mortality than no use of aspirin.

Aspirin in cancer treatment

Aspirin is emerging as a potential novel treatment for patients diagnosed with malignancies, particularly CRC [50]. Several studies have highlighted aspirin’s therapeutic effects in CRC [51-53]. A pivotal study showed that regular aspirin use after a CRC diagnosis significantly reduced cancer-specific and overall mortality [52]. In patients with tumors overexpressing COX-2, aspirin reduced CRC-specific mortality by 29% and overall mortality by 21% [52,54]. Another study found that aspirin use was associated with a decrease in cancer-related death, especially in human leukocyte antigen (HLA) class I antigen-positive patients [55]. This study supported previous findings and emphasized aspirin’s role as an adjunct therapy in specific patient subgroups [56]. Additionally, aspirin has been recognized for reducing recurrence rates and improving survival. Evidence suggests that aspirin reduces mortality and morbidity in cancers beyond colorectal malignancies, including BC and PCa [57].

Recent genome-wide studies have shown a decreased risk of colon cancer in aspirin users, although results vary based on genetic variations at two single nucleotide polymorphisms on chromosomes 12 and 15 [58]. However, evidence suggests that aspirin use in cancer patients may also increase the risk of gastrointestinal and cerebrovascular bleeding, necessitating caution [59]. While low-dose aspirin is effective in cancer prevention, the benefits must be weighed against the significant risk of gastrointestinal bleeding, which, although dose-dependent and lower than that associated with higher doses, remains a concern [60]. Additionally, there may be delays in the onset of aspirin's anti-cancer effects and variability in its metabolism among individuals. Research is ongoing to determine the optimal dose and duration for its efficacy [61]. It is crucial to identify patients who could genuinely benefit from regular aspirin use to help prevent cancers such as CRC in such patients [62]. Lastly, the appropriate age for initiating aspirin therapy to maximize benefits is still under investigation and requires further scrutiny [63].

Potential biomarkers

Identifying biomarkers that predict how cancers, particularly CRC, respond to aspirin therapy is crucial and warrants intensive research. Prostaglandin-endoperoxide synthase 2 (PTGS2), also known as COX-2, plays a significant role in inflammation and tumorigenesis pathways. Studies in mice and colon carcinoma cell cultures have shown increased expression of PTGS2 (prostaglandin G/H synthase and cyclooxygenase) and PGE2 synthase [64]. Aspirin's inhibition of COX-2 has been linked to reduced CRC occurrence. Still, this effect is evident only in patients with high PTGS2 expression in their colonic mucosa, not those with low PTGS2 expression [65]. Therefore, PTGS2 could serve as a valuable biomarker to identify patients more likely to benefit from aspirin therapy, making it a critical factor in personalizing treatment strategies.

Additionally, increased PI3K signaling pathway activity is associated with worse outcomes and treatment resistance in cancer patients. Approximately 15-20% of CRC patients have PIK3CA (phosphatidylinositol-4,5-bisphosphate 3-kinase catalytic subunit alpha) mutation, indicating a potential interaction between aspirin therapy and the PI3K/AKT pathway [66]. Evidence suggests that aspirin’s inhibition of PTGS2 downregulates PI3K signaling activity [67]. Recent studies indicate that CRC patients with PIK3CA mutations experienced a significant decrease in mortality when treated with aspirin, whereas no survival benefit was observed in cases with wild-type PIK3CA [68]. Another study found that aspirin at physiologically attainable levels has a strong anti-cancer effect in those with a PIK3CA mutation compared to the wild type [69]. These findings highlight the potential of using PIK3CA mutation status to identify patients who could benefit substantially from aspirin therapy.

**Figure 1:**
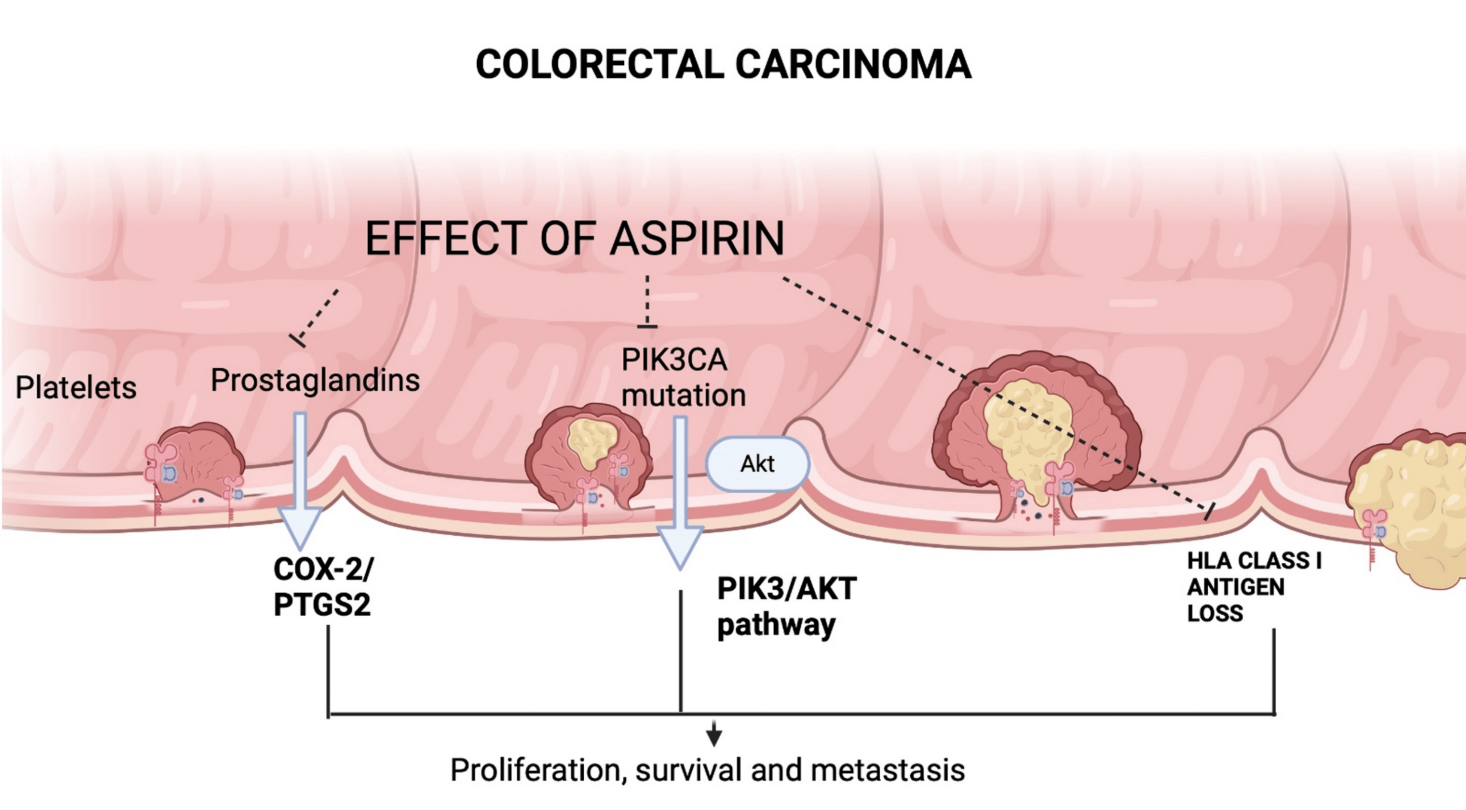
Potential mechanisms of aspirin in colorectal cancer management This figure illustrates the pathways through which aspirin may influence colorectal cancer proliferation and development. Fundamental mechanisms include COX-2 inhibition, modulation of prostaglandins, effects on PIK3CA mutations, and decreased HLA class I antigen expression. Aspirin’s impact on these pathways may lead to reduced cancer cell proliferation, improved survival rates, and lower metastatic potential, underscoring its potential role in cancer therapy, diagnostics, and prevention. Akt: Protein kinase b; COX-2: Cyclooxygenase enzyme-2; HLA: Human leukocyte antigen; PIK3/AKT: Phosphatidylinositol 3-kinase/protein kinase B; PIK3CA: Phosphatidylinositol-4,5-bisphosphate 3-kinase catalytic subunit alpha; PTGS2: Prostaglandin-endoperoxide synthase Image Credit: Author Akash Rawat created on www.biorender.com [71]

HLA class I antigens play a crucial role in immune surveillance and the presentation of tumor antigens to cytotoxic T cells, making their expression in tumor cells a potential biomarker [70]. Recent research also signals that aspirin might influence immunomodulation, particularly in certain HLA class I antigen profiles, thereby bridging its role with immunotherapy [55]. Evidence suggests that aspirin’s immunomodulatory effects might contribute to its anti-cancer properties. For example, aspirin positively impacted CRC patients with high HLA class I antigen expression when administered post-diagnosis and -HLA identification [55]. Thus, HLA class I antigen expression could serve as a valuable biomarker for optimizing aspirin therapy and its combination with immunotherapy. These biomarkers are summarized in Figure 1. 

Future research should explore additional markers in the inflammation cascade, tumor cells, genetic morphology, and epigenetics to predict responses to aspirin therapy. Developing a comprehensive biomarker panel that includes PTGS2, PIK3CA, and HLA class I antigens could revolutionize the duration, dosing, and customization of aspirin therapy for cancer patients. Integrating these biomarkers into routine clinical practice could enhance the personalization of treatment strategies, potentially improving patient outcomes.

Therapeutic interactions

Understanding the interaction of aspirin with other chemotherapeutic or immunotherapeutic agents is crucial for optimizing therapeutic effects and minimizing adverse outcomes. Recent research indicates that aspirin might enhance the potency of current chemotherapy drugs. Its anti-inflammatory properties could counteract the inflammatory processes promoted by tumor cells by inhibiting COX-1, which is normally expressed in colon cells, and COX-2, which is highly expressed in cancers [72]. By reducing the levels of these inflammatory mediators, the inhibition may disrupt the tumor microenvironment, making cancer cells more susceptible to chemotherapeutic agents. Additionally, a systematic review suggested that combining aspirin’s anti-inflammatory effects with effective chemotherapy might reduce tumor growth in mice [73]. Studies have found that combining aspirin with chemotherapy can improve survival outcomes in certain cancers, such as CRC [74].

Moreover, recent evidence suggests that aspirin could enhance the effectiveness of immunotherapy by modifying the cancer cell microenvironment [75]. A retrospective study revealed that using aspirin in conjunction with immunotherapy can improve outcomes by altering the immunological background of tumor cells and making them more susceptible to immunotherapy [75]. Another study found that aspirin might boost immune responses and the efficacy of immune checkpoint inhibitors in patients with malignancies [76].

Aspirin also shows promise in enhancing radiotherapy outcomes. Research indicates that aspirin might increase the radiosensitivity of cervical cancer squamous cells, leading to improved radiotherapy results [77]. Additionally, combining aspirin with radiotherapy could enhance cancer treatment efficacy by promoting tumor cell apoptosis [78].

Although aspirin might enhance certain therapies, its combination with other treatments raises concerns about increased toxicity [15]. For example, one study found that adding aspirin to radiation therapy could lead to greater gastrointestinal (GI) toxicity, bleeding, and mortality [79]. Conversely, another study reported no significant difference in toxicity when aspirin was added to standard treatment [80]. Furthermore, aspirin might acetylate fibrinogen-like protein one and cause increased immunity against tumors and better survival outcomes, suggesting alternative combination immunotherapy in HCC [81]. This highlights the need for careful consideration when integrating aspirin into cancer treatment regimens. Understanding these interactions is essential for effectively optimizing cancer treatment strategies.

Bleeding risks

While aspirin significantly inhibits tumor growth and spread, it has risks. As an irreversible inhibitor of COX-1 and COX-2, aspirin affects TXA2 formation, leading to a higher risk of bleeding [82]. A recent study investigated GI bleeds and hemorrhagic strokes in the elderly, the population most studied with colorectal and intestinal cancers. The study concluded that every two to three bleeds could be attributed to aspirin use, with the remainder having spontaneous causes [5]. Similarly, aspirin use increased intracerebral bleeding by 50% [83]. The well-known study on aspirin in reducing events in the elderly reported a hemorrhage risk of 8.6 events per 1,000 person-years for those taking aspirin compared to placebo. However, the risk of fatal bleeding was reduced to one event per 1,000 person-years [84]. Further evidence indicates that aspirin may increase lower GI bleeding in CRC patients, with a higher absolute risk for new aspirin users compared to non-users three months after starting treatment [85]. Measures to reduce bleeding risk, such as the concomitant use of tranexamic acid, proven in other contexts to reduce bleeding, do not show a statistically significant difference in aspirin patients [86-88]. 

Recent studies have also highlighted a correlation between aspirin use and an increased need for blood transfusions [89]. This association is significant because it can elevate the risk of adverse events related to transfusions, such as allergic reactions or transfusion-related lung injuries. Consequently, it is crucial to closely monitor patients on aspirin for potential immunological reactions and other transfusion-related complications [89].

In addition to these risks, diagnostic and therapeutic interventions for cancer, such as transrectal ultrasound-guided prostate biopsy, also carry a notable risk of bleeding due to their invasive nature. Despite this, recent analyses have shown that the use of aspirin does not significantly increase the bleeding risk associated with prostate biopsies [90]. This finding can be attributed to factors such as the expertise of the healthcare professional performing the procedure and the sample size of the studies reviewed [91,92]. These factors underscore that while bleeding risk is a crucial consideration, the role of aspirin may be less concerning than previously thought for such procedures.

Further imaging and routine complete blood count (CBC) blood tests are recommended to manage the bleeding risk while benefiting from aspirin’s efficacy against tumors. Research has shown that aspirin use can lead to bleeding from premalignant polyps, necessitating colonoscopies, and polypectomies to mitigate this risk. Additionally, aspirin may exacerbate peptic ulcer bleeding, requiring a careful balance between its tumor-restricting and anti-platelet properties. By inhibiting COX-1 and COX-2, aspirin decreases prostaglandins that normally increase blood flow and aid in mucus and bicarbonate formation [93]. The stomach's acidic environment allows aspirin to remain nonionized, leading to increased mucosal permeability. A combination of cellulose-coated aspirin and a proton pump inhibitor (PPI) is recommended to reduce aspirin-induced ulcers. Evidence indicates that combining aspirin with esomeprazole can reduce the incidence of peptic ulcer bleeding compared to a placebo [93].

Additional studies have examined cerebrovascular bleeding risks associated with aspirin. One study found more incidents of spontaneous intracranial bleeding than hemorrhagic strokes, with subdural and subarachnoid hemorrhages being the most common due to trauma [84,94]. Aspirin offers both protective benefits and risks, as summarized in Table 2 [43,57,94-98]. At the same time, it helps prevent myocardial infarction (MI) and tumorigenesis, it also poses risks of GI and intracranial bleeding, particularly from falls or trauma [58].

**Table 2:** Benefits and risks of long-term aspirin use ACEi: Angiotensin-converting enzyme inhibitor; CCB: Calcium channel blockers; H2: Histamine-2 receptor; HR: Hazard ratio; PPI: Proton pump inhibitors; RBC: Red blood cells; TRUS: Transrectal ultrasound-guided scan

Cancer type	Risk reduction of cancer	Risk type	Incidence (HR) of risk type	Recommendations
Colorectal cancer ​[96]​	2.8%	GI bleeding ​[98]​	HR 1.62 (95% CI: 1.25-2.10)	Discontinue aspirin and initiate PPIs (esomeprazole).
Breast cancer ​[57]​	1.7%	Hemorrhagic stroke ​[94]​	HR 1.33 (95% CI: 0.87-2.04)	Discontinue aspirin and initiate fluids and CCBs, along with vessel clamp.
Prostate cancer ​[57]​	1.3%	Intracerebral hemorrhage ​[94]​	HR 1.38 (95% CI: 1.03-1.84)	Discontinue aspirin and initiate fluids and CCBs.
Esophageal cancer ​[57]​	2.3%	Bleeding in therapeutic procedures: TRUS-guided biopsy ​[97]​	HR 1.36 (95% CI: 1.13-1.64)	Discontinue aspirin, administer balloon-catheter tamponade with fluids and total packed RBCs.
Pancreatic cancer ​[57]​	1.9%	Peptic ulcer ​[96]​	HR 1.36 (95% CI: 1.24-1.50)	Discontinue aspirin and use PPIs (esomeprazole) and H2 receptor antagonists.
Hepatocellular cancer ​[43]​	30%	Renal disease ​[95]​	HR 1.54 (95% CI: 1.30–1.82)	Discontinue aspirin and initiate diuretics and ACEi.

Guidelines and recommendations 

The United States Preventive Services Task Force (USPSTF) guidance from April 2016 recommends low-dose aspirin for the primary prevention of cardiovascular disease (CVD) and CRC in adults aged 50 to 59 years [99]. The task force found insufficient evidence to make recommendations for individuals under 50 or over 70. For those aged 60 to 69, the decision to use aspirin should be individualized. However, recent USPSTF guidance from April 2022 revises this stance [100]. It continues to recommend low-dose aspirin for primary CVD prevention but indicates that evidence regarding its effect on CRC incidence and mortality remains unclear.

Limitations

Variability in aspirin dosage, treatment duration, and study methodologies contributes to inconsistent findings. While aspirin benefits CRC, its impact on other cancers remains uncertain [31,32]. Prolonged use is associated with risks, including GI bleeding, intracranial bleeding, and ulcers. The risk-benefit ratio varies among individuals, and safety concerns must be carefully evaluated, particularly for populations at higher risk of complications [57,96]. For this reason, educating the patients about the risks and benefits is important, along with an in-depth understanding of the mechanism by the medical providers to merge efforts to elucidate the risks and benefits of aspirin for cancer according to the actual needs [16,101].

Recommendations

Personalizing aspirin treatment by considering genetic markers and individual risk factors can enhance its efficacy and safety. Large-scale, long-term studies are crucial for more accurately evaluating the risk-benefit profile of aspirin and developing strategies to mitigate adverse effects.

## Conclusions

Aspirin demonstrates considerable potential as both a preventive and a therapeutic agent in cancer management, particularly for CRC. Its primary mechanism involves inhibiting COX-2, reducing prostaglandin levels, and subsequently diminishing cancer-related signaling pathways and inflammation. This action has been shown to lower CRC incidence, mortality, and recurrence rates, with benefits extending to other cancers like BC, PCa, and esophageal cancer. However, variability in efficacy underscores the need for individualized patient assessment to balance aspirin’s benefits with potential risks, such as GI and intracerebral bleeding.

Future research on aspirin’s role in cancer treatment should focus on refining its therapeutic window by determining optimal dosing regimens and treatment durations to maximize efficacy while minimizing adverse effects. In-depth studies are necessary to identify and validate biomarkers that predict which patients are most likely to benefit from aspirin therapy, enabling personalized treatment strategies. Additionally, it is crucial to explore aspirin’s interactions with other treatments, including chemotherapy, immunotherapy, and radiotherapy, to optimize combination therapies. Long-term epidemiological studies are essential to track the effects of aspirin use across diverse cancer populations and evaluate its impact on survival, quality of life, and cancer recurrence rates. Addressing these research gaps will enhance our understanding of aspirin’s role in cancer therapy and contribute to the development of safer, more effective treatment strategies.
